# Trans-scapular approach coil localization for scapular-blocked pulmonary nodules: a retrospective study

**DOI:** 10.1186/s13019-021-01446-6

**Published:** 2021-03-25

**Authors:** Juan Wu, Min-Ge Zhang, Jin Chen, Wen-Bin Ji

**Affiliations:** Department of Radiology, Taizhou Hospital of Zhejiang Province affiliated to Wenzhou Medical University, Taizhou, China

**Keywords:** Computed tomography, Scapular, Coil, Pulmonary nodule

## Abstract

**Background:**

Preoperative computed tomography (CT)-guided coil localization (CL) is commonly used to facilitate video-assisted thoracoscopic surgery (VATS)-guided diagnostic wedge resection (WR) of pulmonary nodules (PNs). When a scapular-blocked PN (SBPN) is localized, the trans-scapular CL (TSCL) is commonly performed. In this study, we investigated the safety, feasibility, and clinical efficacy of preoperative CT-guided TSCL for SBPNs.

**Materials and methods:**

From January 2014 to September 2020, a total of 152 patients with PNs underwent CT-guided CL prior to VATS-guided WR. Of these patients, 14 had SBPNs and underwent the TSCL procedure.

**Results:**

A total of 14 SBPNs were localized in the 14 patients. The mean diameter of the 14 SBPNs was 7.4 ± 2.4 mm. The technical success rate of the scapula puncture was 100%. No complications occurred near the scapula. The technical success rate of CL was 92.9%. One coil dropped off when performing the VATS procedure. The mean duration of the TSCL was 14.2 ± 2.7 min. Two patients (14.3%) developed asymptomatic pneumothorax after TSCL. The technical success rate of VATS-guided WR was 92.9%. The patient who experienced technical failure of TSCL directly underwent lobectomy. The mean duration of the VATS was 90.0 ± 42.4 min and the mean blood loss was 62.9 ± 37.2 ml. The final diagnoses of the 14 SBPNs included invasive adenocarcinoma (*n* = 4), adenocarcinoma in situ (*n* = 9), and benign disease (*n* = 1).

**Conclusions:**

Preoperative CT-guided TSCL is a safe and simple procedure that can facilitate high success rates of VATS-guided WR of SBPNs.

## Background

Pulmonary nodules (PNs) are commonly detected in the clinic by chest computed tomography (CT) [1–5]. Although most of PNs can be regularly followed up by CT according to the Fleischner Society or Lung-RADS guidelines [6, 7], many intermediate or high risk PNs should be managed actively. Currently, lung biopsy or video-assisted thoracoscopic surgery (VATS)-guided diagnostic wedge resection (WR) are widely used to diagnose the PNs [8–10]. Although lung biopsy is a minimally invasive procedure that requires only local anaesthesia, it is associated with a diagnostic failure rate of around 10% [11].

Preoperative CT-guided localization is commonly used to facilitate the VATS-guided diagnostic WR of PNs as it decreases the need for thoracotomy or VATS anatomic resection for the diagnosis of PNs [8, 12]. Localized materials usually include coils, hook-wire, methylene blue, and radio-labeling agents [8]. The process of coil localization (CL) typically has the lowest rate of complications [8].

Although most of the PNs can be easily detected with a needle pathway to perform the biopsy or localization, some PNs can be blocked by the scapula [13, 14]. Under the typical conditions, the scapula can be punctured when performing the lung interventions for the scapula-blocked lung lesions [13, 14]. However, few studies have reported on trans-scapular CL (TSCL) for scapular-blocked PNs (SBPNs).

In this study, we aimed to investigate the safety, feasibility, and clinical efficacy of preoperative CT-guided TSCL for SBPNs.

## Methods

This retrospective, single-center study was approved by our Institutional Review Board and did not require written informed.

### Study design

From January 2014 to September 2020, a total of 152 patients with PNs underwent CT-guided CL prior to VATS-guided WR. Of these patients, 14 patients (9.2%) had SBPNs and underwent the TSCL procedure.

The inclusion criteria were as follows: (a) a definite SBPN detected on CT; (b) PNs with a diameter ≤ 3 cm (sub-solid PNs ≤ 30 mm; solid PNs ≤ 15 mm); (c) the PN-pleura distance ≤20 mm; and (d) PNs lacking a definite pathological diagnosis.

The exclusion criteria were as follows: (a) a PN diameter < 5 mm; (b) a PN which decreased in size during CT follow-up; and (c) any abnormal coagulation activity, active bleeding, active infections, or limited cardiopulmonary reserve.

### Puncture of the scapula

All procedures were performed by an interventional radiologist with more than 5 years of experience conducting CT-guided interventions using a 64-row CT (GE Healthcare, Milwaukee, WI). Patients were placed in the prone position and were administered local anesthesia.

**Fig. 1:**
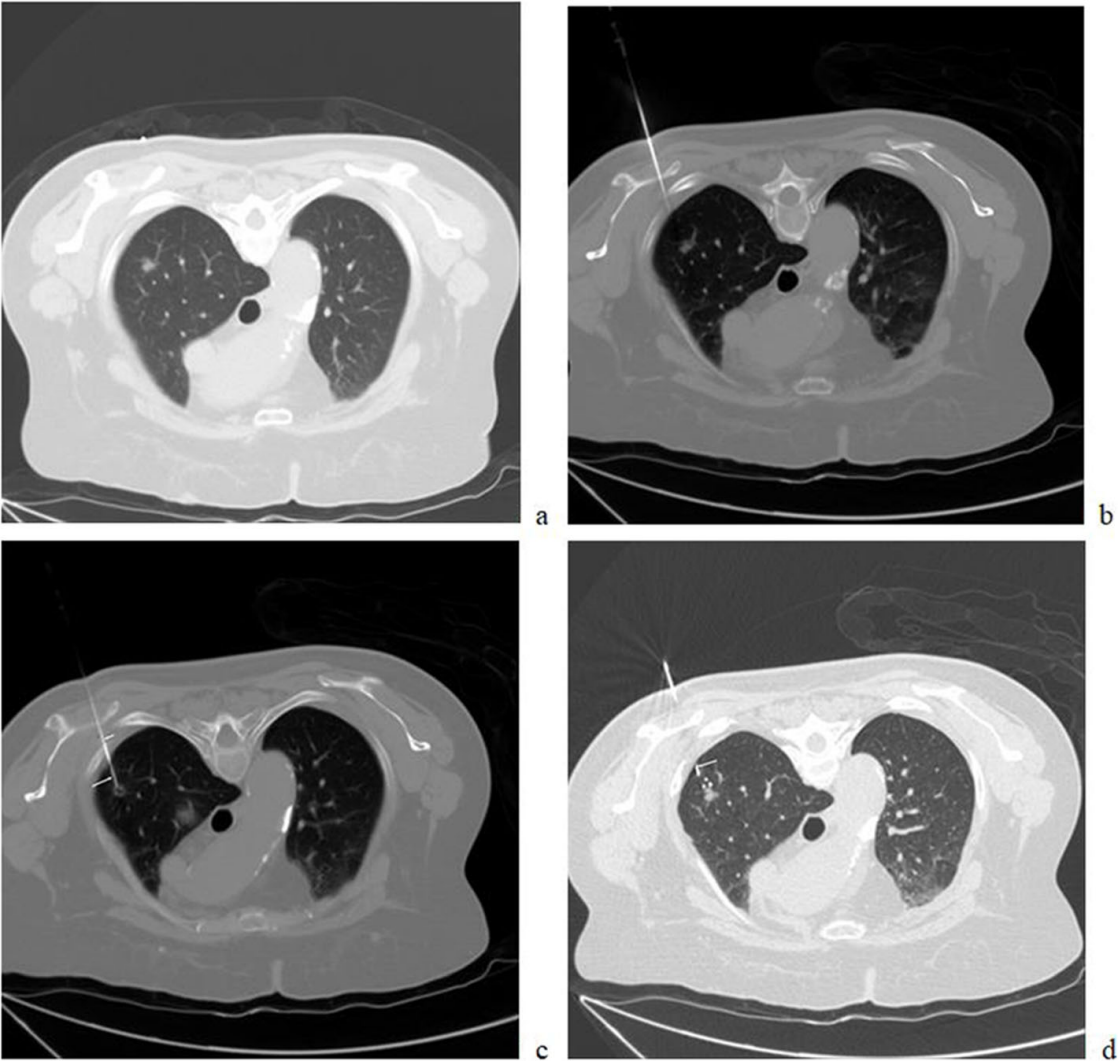
(**a**) Preoperative CT indicating a SBPN located at the right upper lobe; (**b**) A 17G needle was used to puncture the scapular; (**c**) An 18G needle (long arrow) was inserted into the lung for coil localization via the 17G needle (short arrow); (**d**) The coil tail (arrow) remained above the visceral pleura

A preoperative CT scan was used to ensure the needle pathway (Fig. 1a). A 17G needle (DuoSmart, Modena, Italy) was used to puncture the scapula. When the needle contacted the scapula, it was punctured using a drill and inserted under steady pressure. A repeat CT scan was performed to observe the location of the needle tip and any procedure-related complications (Fig. 1b). When the 17G needle was passed across the scapula, the needle tip was carefully pushed close to the lung, but the needle tip did not enter the lung.

### Localization procedure

When the 17G needle was passed through the scapula, an 18G needle (Precisa, Roma, Italy) was inserted from the 17G needle and smoothly pushed to the lung to within approximately 10 mm of the PN (Fig. 1c). Next, a coil (5 cm long and 0.038 in. in diameter, Cook, Bloomington, IN) was partially placed into the pulmonary parenchyma. The needle was smoothly retracted to ensure that the coil tail remained above the visceral pleura (Fig. 1d). A repeat CT scan was performed to observe the location of the coil and any procedure-related complications.

### VATS procedure

VATS-guided WR was routinely performed within 24 h of localization. The coil tail was used to guide this procedure. When the coil tail was detected under the thoracoscope, the WR was performed with a cutting margin > 20 mm from the coil tail. If the coil tail was not visible, the coil was considered to be completely inserted into the pulmonary parenchyma. Palpation of the coil was performed to conduct the WR. If this procedure is unsuccessful, lobectomy should be performed.

The resected lesions were sent for a rapid pathological examination. If the pathological diagnosis indicated a benign lesion, carcinoma in situ, mini-invasive carcinoma, or metastatic PN, the VATS was terminated. In these cases, further lobectomy and lymph node dissection should be performed when the PN is diagnosed as invasive carcinoma.

### Definitions

SBPN was defined as the PN with the lesion-pleura vertical line striding across the scapula. The technical success of TSCL was defined when the coil tail could be detected under the thoracoscope. The technical success of the WR was defined when the PN was found in the resected wedge tissue.

The primary endpoint was the technical success of the TSCL. The secondary endpoints included localization-related complications, technical success of VATS-guided WR, and final diagnoses of the PNs.

### Statistical analysis

All statistical analyses were conducted using SPSS 16.0 (SPSS Inc., Chicago, IL). Continuous variables are presented as the mean ± standard deviation. Categorical data are presented as a percentage (number/total).

## Results

### Patients

The baseline data of the 14 patients are summarised in Table 1. Seven females and 7 males were analysed in the study and the patients had a mean age of 57.0 ± 7.8 y. None of the patients had a previous history of cancer.

**Table 1:** Baseline data of the 14 patients

	Values
Patients number	14
Age (y)	57.0 ± 7.8
Gender (male/female)	7/7
Tumor history	0
Smoking history	5

### SBPNs

The baseline data of the SBPNs are shown in Table 2. Each patient had 1 SBPN. The mean diameter of the 14 SBPNs was 7.4 ± 2.4 mm. Eight SBPNs were located in the upper right lobe and 6 were located in the upper left lobes.

**Table 2:** Baseline data of the nodules

	Values
Nodules number	14
Diameter (mm)	7.4 ± 2.4
Natures of nodules	
Solid	6
Sub-solid	8
Nodule-pleura distance (mm)	5.6 ± 5.1
Side	
Left	6
Right	8

The indications for resection of the SBPNs included: (a) high-risk of lung cancer based on the clinical-radiological features (*n* = 8) [1, 6, 7]; and (b) PNs with the increasing size or solid components (*n* = 6).

### TSCL procedure

The technical success rate of puncture of the scapula was 100% and no complications occurred near the scapula. The technical success rate of CL was 92.9% (13/14, Table 3). One coil dropped off when performing the VATS procedure. The mean needle-pleura degree was 80.4 ± 4.5 degrees. The mean duration of the TSCL was 14.2 ± 2.7 min.

**Table 3:** Details of CT-guided localization

	Values
Technical success of localization	13 (92.9%)
Duration of CT-guided procedure (min)	14.2 ± 2.7
Needle-pleura degree	80.4 ± 4.5
Complications	
Haematoma near the scapula	0
Pneumothorax	2 (14.3%)
Lung haemorrhage	0

*CT* computed tomography

Two patient (14.3%) developed aysmptomatic pneumothorax after TSCL but this did not impact the subsequent VATS procedure.

### VATS-guided WR

The technical success rate of the VATS-guided WR was 92.9% (13/14, Table 4). The patient who experienced technical failure of the TSCL directly underwent lobectomy. Four patients underwent additional lobectomy after WR due to the pathological diagnosis of invasive adenocarcinoma.

**Table 4:** Details of VATS procedures

	Values
Technical success of WR	13 (92.9%)
Types of surgery	
WR	9
WR + lobectomy	4
Lobectomy	1
Duration of VATS (min)	90.0 ± 42.4
Blood loss (ml)	62.9 ± 37.2
Final diagnoses	
Invasive adenocarcinoma	4
Adenocarcinoma in situ	9
Benign	1

*VATS* video-assisted thoracoscopic surgery; *WR* wedge resection

The mean duration of the VATS procedure was 90.0 ± 42.4 min and the mean blood loss was 62.9 ± 37.2 ml. The final diagnoses of the 14 SBPNs included invasive adenocarcinoma (*n* = 4), adenocarcinoma in situ (*n* = 9), and benign disease (*n* = 1). The pathological diagnosis of the SBPN in the patient who experienced technical failure WR was adenocarcinoma in situ.

## Discussion

The present study demonstrated the feasibility, safety, and clinical efficacy of preoperative CT-guided TSCL for SBPNs. During the CT-guided lung interventions, the needle pathway may be blocked by the bone structures [13, 14]. In most cases, the bone structures should be avoided and an alternative pathway chosen. However, unlike other lung interventions (biopsy or ablation), CT-guided CL of PNs requires the coil tail to remain on the pleural surface nearest the PN to ensure the technical success of VATS-guided WR [12]. Thus, the shortest needle pathway is essential to the CT-guided CL procedure even when the optimal pathway is blocked by the bone structures.

In the current study, the scapula was successfully punctured in all patients with a high technical success rate (92.9%) of CT-guided TSCL. These rates were consistent with technical success rates previously reported in studies of CT-guided trans-bone lung ablation or biopsy (91–100%) [13–15] and in CT-guided CL for PNs (90–100%) [12, 16–18]. Previous reports of percutaneous needle approaches in the scapular region did not observe neurovascular injury or significant hemorrhage associated with the procedures [13–15]. In this study, no complications were observed near the scapula.

Several previous clinical studies have reported on the application of CT-guided CL for sub-fissural or multiple PNs [18–20]. In comparison to these studies, the CT-guided TSCL technique is simpler as it only necessitates puncturing of the scapula and we found that this could be achieved effectively using a 17G needle. Following the puncture of the scapular, an 18G needle was coaxially inserted to facilitate coil placement and to reduce the risk of pneumothorax.

In this study, the rate of asymptomatic pneumothorax was 14.3% which is comparable to that observed in previous studies of CT-guided coil localization (9–40%) [16–18] and CT-guided trans-scapular lung interventions (18–29%) [13–15].

In this study, the technical success rate of VATS-guided WR was 92.9% which is consistent with findings for the majority of preoperative CT-guided CL in other special PNs (95–100%) [18–20]. Preoperative CT-guided TSCL can achieve a high technical success rate of WR when evaluating SBPNs and also preserve maxim lung function .

Our study had several limitations. The analysis was performed as a retrospective study from a single-center and is subject to selection bias and the sample size was small. However, our study focused on special cases that had SBPNs. Previous studies that have focused on trans-scapula lung interventions have also analyzed small patient cohorts limited to 5–12 patients [13–15]. Also, no control group was set in this study. When we began to use the preoperative localization for PNs, we always used the CLand had no cases that were localized by other materials. Further randomized controlled trials are needed to validate our findings.

## Conclusions

Preoperative CT-guided TSCL can be safely and simply used to facilitate high success rates of VATS-guided WR of SBPNs.

## Data Availability

The data that support the findings of this study are available from the corresponding author upon reasonable request.

## Abbreviations

CT: Computed tomography
CL: Coil localization
PN: Pulmonary nodule
SBPN: Scapular-blocked PN
TSCL: Trans-scapular CL
VATS: Video-assisted thoracoscopic surgery
WR: Wedge resection
